# Deep learning‐based classification and mutation prediction from histopathological images of hepatocellular carcinoma

**DOI:** 10.1002/ctm2.102

**Published:** 2020-06-14

**Authors:** Haotian Liao, Yuxi Long, Ruijiang Han, Wei Wang, Lin Xu, Mingheng Liao, Zhen Zhang, Zhenru Wu, Xuequn Shang, Xuefeng Li, Jiajie Peng, Kefei Yuan, Yong Zeng

**Affiliations:** ^1^ Department of Liver Surgery & Liver Transplantation, State Key Laboratory of Biotherapy and Cancer Center, West China Hospital Sichuan University and Collaborative Innovation Center of Biotherapy Chengdu China; ^2^ School of Computer Science Northwestern Polytechnical University Xi'an China; ^3^ Key Laboratory of Big Data Storage and Management Northwestern Polytechnical University, Ministry of Industry and Information Technology Xi'an China; ^4^ Department of Radiology, West China Hospital Sichuan University Chengdu China; ^5^ Laboratory of Pathology Department of Pathology, West China Hospital Sichuan University Chengdu China; ^6^ School of Basic Medical Sciences Guangzhou Medical University Guangzhou China; ^7^ Shenzhen Luohu People's Hospital The Third Affiliated Hospital of Shenzhen University Shenzhen China

**Keywords:** artificial Intelligence, convolutional neural network, hepatocellular carcinoma, pathology

Despite of the fact that the diagnosis of hepatocellular carcinoma (HCC) mainly relies on noninvasive approaches including computerized tomography or magnetic resonance imaging,^1^, ^2^ evaluation of histopathology is still indispensable in the clinical care of patients, as pathology can not only allow for a definitive diagnosis but also provide significant prognostication information.^3^ Moreover, histological subtypes of HCC have been shown to be related to somatic mutation burdens,^3^, ^4^ which suggests the link between HCC molecular features and histological phenotypes. Recently, the association between the occurrence of activating mutations and the response to multiple tyrosine kinase inhibitors or immunotherapy has been established in HCC patients.^5^, ^6^, ^7^, ^8^ Taken together, these findings support the establishment of personalized management for each HCC patient based on histopathology. However, visual inspection on tissue slides is typically performed at magnifications from 5× to 40× in an exhaustive manner, which makes it time‐consuming for a pathologist to interpret the complexity of histopathological morphology.^9^ In this study, we constructed a convolutional neural network (CNN)‐based platform using whole‐slide images (WSIs) of hematoxylin and eosin (H&E)‐stained digital slides obtained from The Cancer Genome Atlas (TCGA) dataset, as well as HCC tissue microarrays (TMAs) from The Biobank of West China Hospital (WCH), to realize the automatic diagnosis of HCC (task 1) and prediction of somatic mutation (task 2).

**HIGHLIGHTS**

- HCC pathology can not only allow for a definitive diagnosis but also provide significant biological information.
- Deep convolutional neural network using HCC histopathological slides can realize the automatic diagnosis of HCC and somatic mutation prediction.
- The deep learning‐based histopathology may serve as a promising tool to free the pathologists from dull routine practice.

Generally, two datasets of H&E‐stained digital slides were collected in our studies: (a) WSIs of HCC with matched adjacent normal tissues from TCGA dataset (Figure 1A, left panel) and (b) TMAs constructed from 455 HCC samples with 265 matched normal tissues from The Biobank of West China Hospital (Figure 1A, right panel). Primarily, a total of 491 WSIs in TCGA dataset were downloaded from GDC data portal (https://portal.gdc.cancer.gov), which consisted of 402 HCC slides with 89 matched ones of adjacent normal tissue. HCC slides with diagnosis other than HCC and those with readability issues were excluded from further analysis, as well as their matched normal tissue slides. The corresponding clinical and pathological data for each WSI in TCGA dataset were downloaded from The cBioPortal for Cancer Genomics.^10^, ^11^ For the TMA data of WCH dataset, we used the same inclusion strategy as that for TCGA dataset. For the use of TMA samples, each participant in this study provided the informed consent, which was approved by The Ethics Committee of West China Hospital. For the construction of CNN models, we used 80% of the tiles in TCGA dataset for training and 20% for testing (Figure 1B, left panel, Table S1). To prevent overlaps between these two sets, we integrated tiles from the corresponding slide as a whole to one of the sets. The information of somatic mutations in each dataset can be found in the Supporting Information. For each task, we trained a CNN with introducing the structure of deep residual learning^12^ (Methods in the Supporting Information) to overcome the degradation problem, and the probability of each slide or TMA dot was generated using two methods: (a) averaging of the probabilities of tiles from the corresponding slide (generating Method 1) and (b) summarizing the percentage of positively classified tiles from the corresponding slide (≥0.5) (generating Method 2). The architecture of our CNN models can be found in Figure S1 and Methods in the Supporting Information. Before model trainings, each digital slide (WSI or TMA) was preprocessed using OpenSlide library^13^, ^14^ (Methods in the Supporting Information).

**FIGURE 1:**
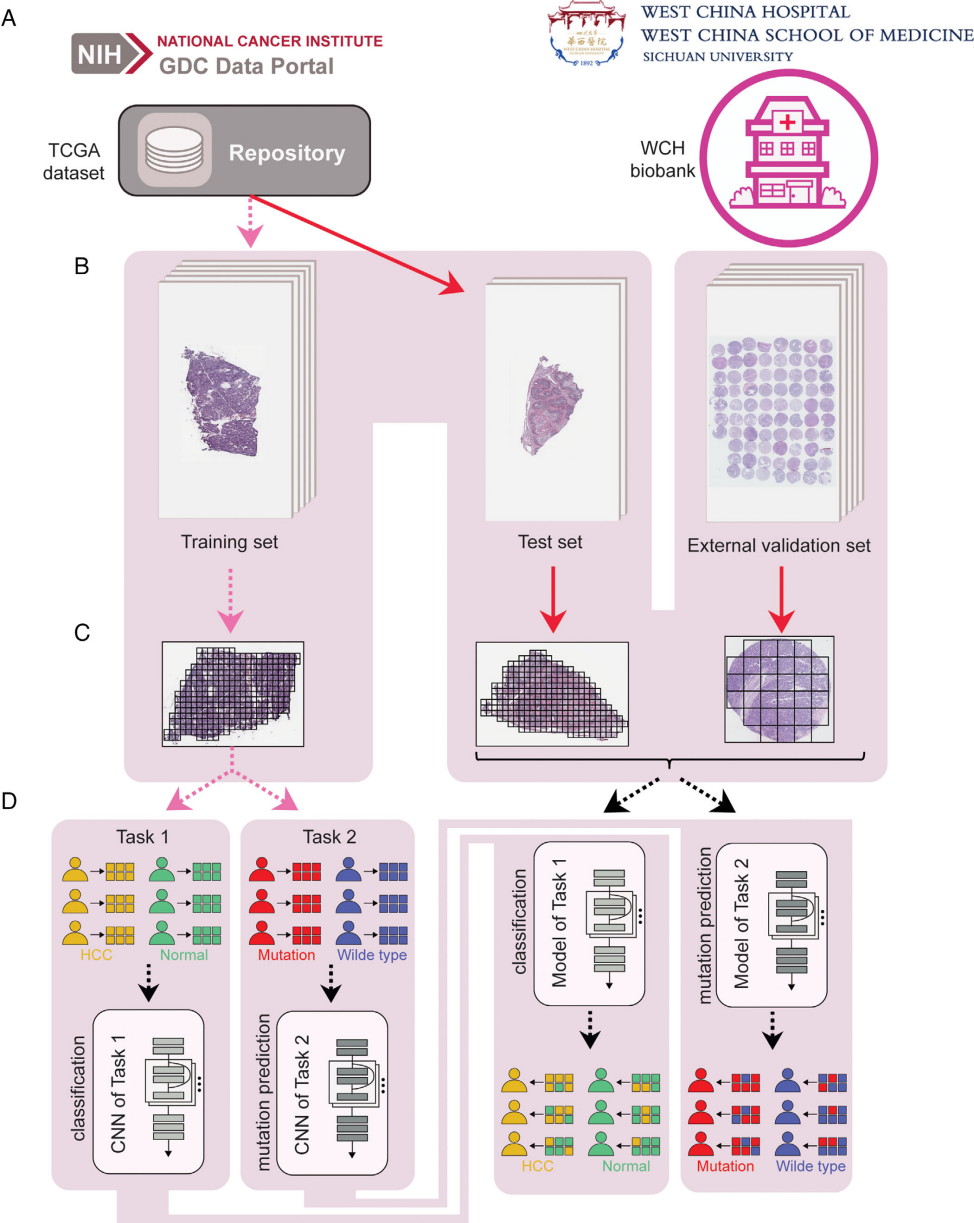
Overview of the data and proposed deep learning framework presented in this study. A, Images used for this study were obtained from the TCGA database (left panel) and the WCH biobank (right panel). B, For the training and test of our CNN models, images from TCGA dataset were divided into a training (80%) and a test set (20%). TMA dots from the WCH biobank consist the whole external validation set. C, For the training, testing, and validation of our models, each slide or TMA dot was tiled into nonoverlapping 256 × 256 pixel patches, and tiles with over 12.5% background were omitted. This process was then followed by the normalization of RGB values. D, Tiles from the training set were used as the input for the both two CNN models (tasks 1 and 2), and the performance of our CNN models was tested on tiles from the test set. To challenge the trained models for tasks 1 and 2 and identify their limitations, both tasks 1 and 2 were also performed on tiles from TMAs

The datasets we used and the whole deep learning strategy were summarized in Figure 1. After excluding slides according to the inclusion criteria, 393 HCC slides and 88 slides of matched normal tissues were recruited in this study (Figure 1A, left panel). Eleven TMA slides from 455 HCC patients in WCH dataset (Figure 1A, right panel), which contained 719 dots including 455 HCC dots and 264 dots of matched normal tissues, were used as the external validation set (Figure 1B, right panel). The whole TCGA dataset was split into two sets: training set (408 slides) for the training of our CNN models (tasks 1 and 2), and test set (73 slides) for the test of the models (Figure 1B, left panel). To accelerate the deep learning‐based computational processes, the CNNs were trained and tested on 256 × 256 pixel tiles (Figures 1C and 1D), which resulted in >950 000 and >120 000 tiles from TCGA dataset and WCH dataset, respectively (Figure S2).

According to the deep learning‐based strategy illustrated in Figure 1, we first developed a classification CNN that can distinguish HCC from adjacent normal tissues (task 1). Details about the number of slides and tiles used in each set for task 1 can be found in Table S1. Histograms showed the distribution of probabilities on HCC and normal tiles for HCC diagnosing (Figure S3). In the test set, less than 10% of tiles were misclassified at a magnification of 5× (8.6%, Figure S3A, right panel) or 20× (6.1%, Figure S3A, left panel), and the per‐tile classification results at both two magnifications yielded an area under the ROC curve (AUC) of over 0.97 (Figure S3B; Table S2). However, in the external validation set, 12.3% and 21.6% of tiles were misclassified at 5× magnification (Figure S3C, left panel) and 20× magnification (Figure S3C, right panel), respectively. Despite this fact, per‐tile classification at 5× magnification (Table S2) also showed an AUC of 0.949, whereas classification at 20× magnification yielded an accuracy that is significantly lower than that at 5× magnification (AUC = 0.860; Figure S3D).

In order to assess the classification accuracy on per‐slide level, the per‐tile classification results were aggregated using the two methods previously described to generate a per‐slide classification. Both generating Methods 1 and 2 resulted in an almost error‐free classification in the test set (Figures 2A and S4A; Table S2). Consistent with the results from per‐tile classification, the AUCs achieved in the external validation set by both two methods at 5× magnification were significantly higher than those at 20× magnification (Figures 2B and S4B; Table S2). Nevertheless, dots labeled with “HCC” still demonstrated significantly higher probabilities of HCC diagnosis at 20× magnification when using the CNN classifier (Figures 2B and S4B, left panel; Table S2). Next, we analyzed the correlation between the results obtained from the two magnifications to investigate the agreement of per‐slide (or per‐dot) classification results achieved at different resolutions (5× vs 20×). It was found that in both the two validation sets (test and external validation), the classification results aggregated at these two magnifications were highly correlated (Figures 2C, 2D, S4C, and S4D). Although high consistency was observed between the results from the two resolutions when using a binary classifier in the test set (Figures 2E and 2G ), ∼50% of the TMA dots assigned with a “Normal” label at 5× magnification showed the opposite classification outcomes when 20× magnified tiles were used (Figures 2F and 2H). It is worth noticing that no significant correlation was found between the accuracy of the classification and the WSI (or TMA) size (Figure S5; Spearman's correlation coefficient <0.5).

**FIGURE 2:**
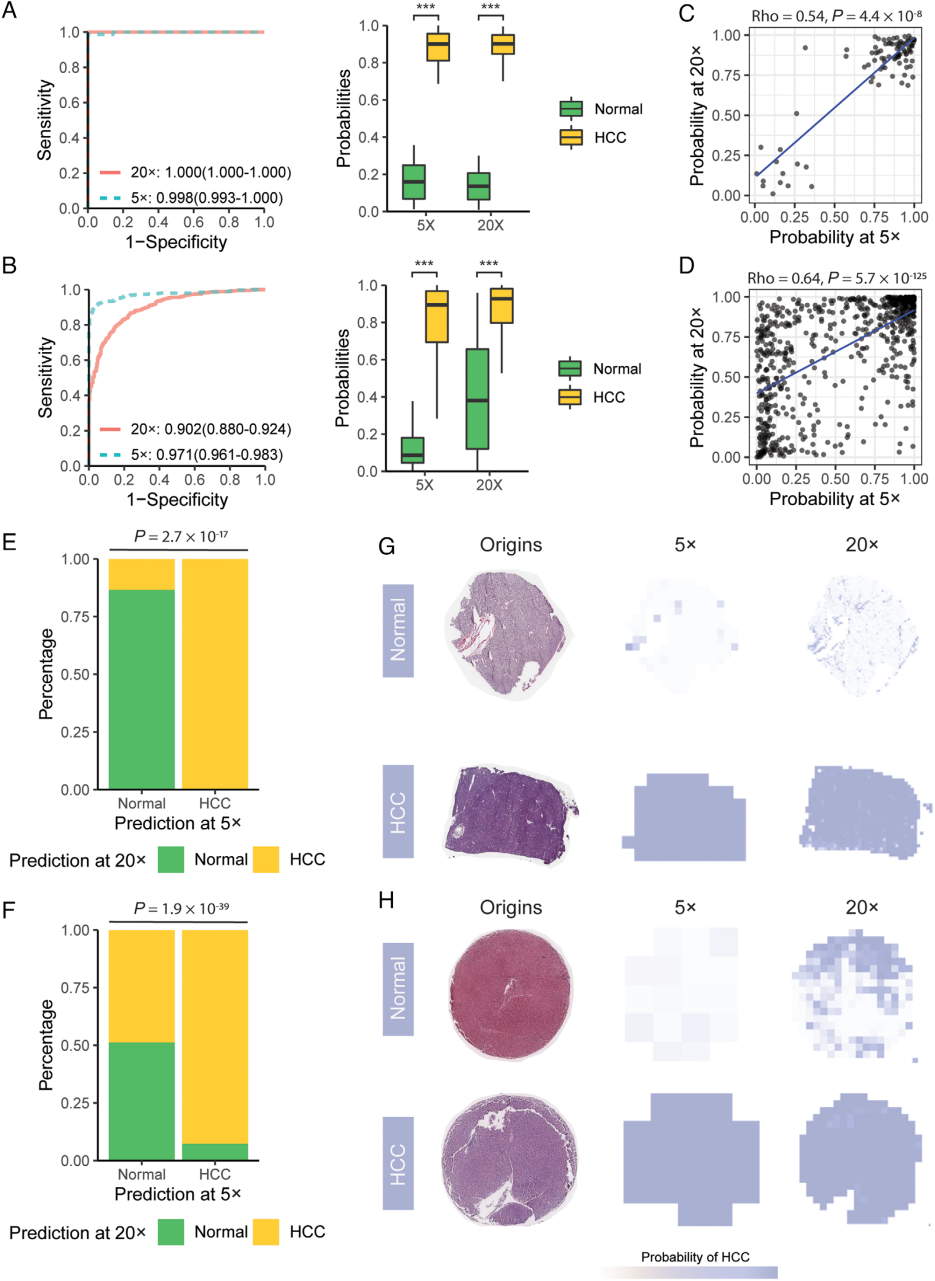
Detection of presence of HCC. A and B, The receiver operating characteristic (ROC) curvesof our diagnosing model (AUC with 95% CI) for distinguishing HCC from adjacent normal tissues in the test set (A, left panel) and external validation set (B, left panel). Box plots (Mann‐Whitney U‐test) demonstrated the probability of HCC diagnosis predicted (right panel). The probability of each slide (or dot) was generated using Method 1. ^***^ *P* < .001. C and D, Scatter plots showing the correlation between the per‐slide classification results obtained at different resolutions (5× vs 20×) in the test set (C) and external validation set (D). The probability of each slide (or dot) was generated using Method 1. E and F, Bar plots demonstrating the consistency in the classification results between two magnifications (5× vs 20×) when using binary classifiers in the test set (E) and external validation set (F). The probability of each slide (or dot) was generated using Method 1. G, A typical example of raw images in the test set with the corresponding heatmaps obtained by the classification CNN. A high consistency between the results obtained at two resolutions (5× vs 20×) was observed. H, A typical example of raw images in the external validation set with the corresponding heatmaps obtained by the classification CNN. Although the dot of normal tissue was recognized at 5× magnification, this dot has been misclassified using 20× magnified tiles

To investigate the capability of CNN in predicting the mutation status of HCC samples by using histopathological images as the only input, gene mutation data for the matched WSIs of HCC samples from TCGA dataset were used in CNN training for task 2 (Methods in the Supporting Information). During the training process, we only selected the mutations with minor allele frequency (MAF) ≥10% among the available tumors in TCGA dataset, which could ensure that both training and test sets had sufficient images from the mutations (Table S3). In addition, the CNN network for task 2 was derived from the modified classification CNN for task 1 by replacing the softmax layer with a sigmoid layer (Methods in the Supporting Information). Only 20×‐magnified tiles were used for this task because 20× is the requisite to extract predictive features for mutation predictions, whereas 5× can only demonstrate a random performance.^9^ The training process was performed on ∼569 000 tiles from the training set, and the established mutation‐prediction model was validated on ∼105 000 tiles from the test set (Table S4). The results from box plot and receiver operating characteristic (ROC) curves showed that the mutation‐prediction CNN could identify predictable mutations in seven genes (defined as AUC ≥ 0.7 and two‐tailed Mann‐Whitney U‐test *P* < .05), including ALB, CSMD3 (CUB and Sushi multiple domains 3), CTNNB1, MUC4, OBSCN (obscurin, cytoskeletal calmodulin, and titin‐interacting RhoGEF), TP53, and RYR2 (ryanodine receptor 2), by using either per‐tile prediction results (Figures S6A and S6B; Table S5) or the aggregation results (Figures 3A, 3B, S7A, and S7B; Table S5). Among these seven genes, AUCs for CTNNB1, which is one of the primary oncogenes involved in HCC development,^15^ reached the highest value at 0.903 (Table S5), indicating that mutations of CTNNB1 in the test set were highly predictive by our CNN model (Figure 3C). It is also noticeable that AUCs for TP53, of which the somatic mutations contribute to human cancers in different ways,^16^, ^17^ reached a value at 0.773 using Method 1 (Table S5). The heatmap showed that slides of TP53‐mutated samples could also be easily distinguished from those of wild‐type samples (Figure 3D). The distribution of probabilities on mutated and wild‐type tiles for these seven predictable mutations in the test set was demonstrated in Figure S8A, in which a higher percentage of positively classified tiles for each mutation in the mutated samples could be easily observed.

**FIGURE 3:**
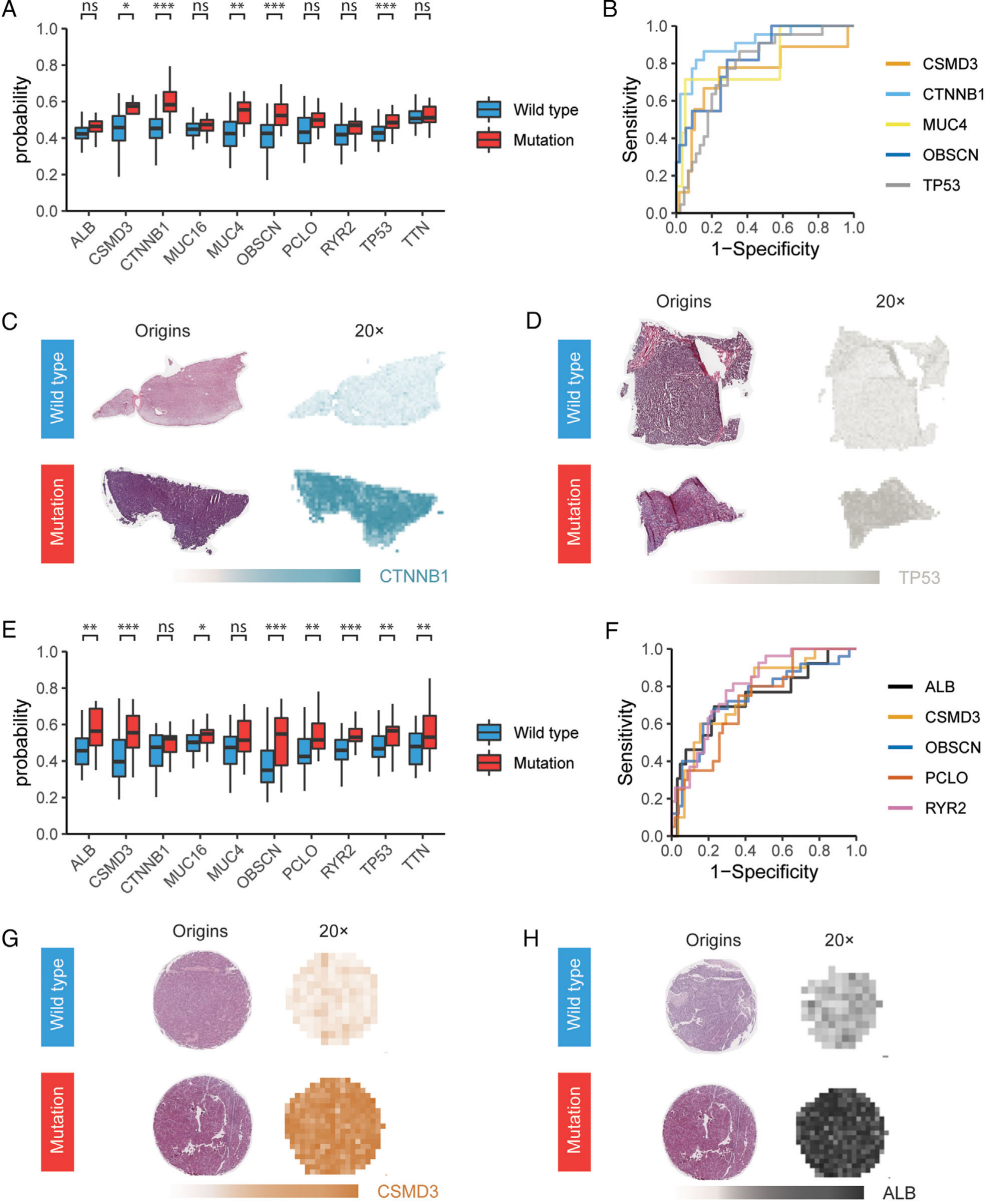
Gene mutation prediction results in both the test and external validation set. A, Probability distribution of mutations from WSIs where each mutation is absent or present in the test set (results aggregated by Method 1). ns, not significant; ^*^ *P* < .05; ^**^ *P* < .01; ^***^ *P* < .001. B, ROC curves of the top 5 predictions in panel (A). C and D, Typical examples of raw images in the test set with the corresponding heatmaps obtained by the mutation‐prediction CNN for CTNNB1 (C) and TP53 (D) predictions in the test set. Significant differences could be observed in the color density between mutated and wild‐type samples. E, Probability distribution of mutations from TMA dots where each mutation is absent or present in the external validation set (results aggregated by Method 1). ns, not significant; ^*^ *P* < .05; ^**^ *P* < .01; ^***^ *P* < .001. F, ROC curves of the top five predictions in panel (E). F and G, Typical examples of raw images in the test set with the corresponding heatmaps obtained by the mutation‐prediction CNN for CSMD3 (F) and ALB (G) predictions in the external validation set. Significant differences could be observed in the color density between mutated and wild‐type samples

For the purpose of challenging the mutation‐prediction CNN and testing its generalizability, we then evaluated this model on TMA dots of HCC samples with available WES data in the external validation set, which consists of 78 TMA dots from 78 samples, and the validation was conducted on over 9800 tiles (Table S4). Among the predictable gene mutations in test set, in four of them, our CNN model exhibited the predictive capability in the external validation set: ALB, CSMD3, OBSCN, and RYR2 (Figures S6C, S6D, 3E, 3F, S7C, and S7D; Table S6). For instance, in both the test set (Table S5) and external validation set (Figure 3G, Table S6), our mutation‐prediction model also successfully identified samples with mutations of CSMD3, which is the second most frequently mutated gene (next to TP53) in lung cancer.^18^ Meanwhile, the mutations of ALB, a key mediator of hepatocyte function in the secretion of blood factors, albumin and VLDL, were also predicted with high AUCs > 0.7 in both the two sets (Figure 3H; Tables S5 and S6). On the other hand, the other two mutations that were not predictable in the test set (TTN and PCLO) could also be predicted in the external validation set (Table S6). However, mutations of CTNNB1 and TP53, which were predicted with high AUCs in the test, could not be predicted at this stage (Table S6). These findings suggested that there were some important differences between WSIs and our TMA dots impacting the evaluation of the TCGA‐based model. Despite this fact, box plot showed significant difference in the probability of HCC diagnosing between TP53‐mutated samples and those wild‐type ones (Figures 3E, S6C, and S7C). The distribution of probabilities on mutated and wild‐type tiles for these four predictable in the external validation set also showed a higher percentage of positively classified tiles for each mutation in the mutated samples than that of wild‐type ones (Figure S8B).

In conclusion, we have provided with a promising perspective on HCC diagnosis using CNN, which unambiguously distinguished tumor from adjacent normal tissues using WSIs (highest AUC achieved at 1.000), which even outperformed the AUC of ∼0.99 achieved in our previous work using image features combined with random forest classifier.^19^ Regarding the performance on TMAs, there was a gain of ∼0.2 in AUC by the CNN model compared to results using feature‐based approach at 20× magnification.^19^ Moreover, compared with Inception V3 model that showed excellent performance on WSIs,^9^ our models cost less memory and time and demonstrated higher prediction accuracy in both tasks 1 and 2 (Table S7; Figures S9 and S10). However, we noticed a significant difference in the results of task 1 between TMA dots at different resolutions (5× vs 20×). This finding might be attributed to the fact that, compared with feature extraction at 5× magnification, more tiles are inundated with some “misleading” features, such as air bubbles, dull staining, and uneven staining during TMA preparation, leading to a more ambiguous per‐tile diagnosis of HCC, which in turn contributed to a more ambiguous per‐dot HCC diagnosing. The discrepancy between the TCGA and WCH dataset using the mutation‐prediction CNN might be owing to the fact that only the most representative view of each sample was used after pathologists browse through each region in WSIs during TMA construction, which might lead to the loss of significant information on the histopathological characteristics of tumor samples. Despite these, we do believe that our work will inspire further studies extending our classification model to the specific histological subtypes of HCC and predicting their genetic alterations. In the future, studies based on a large scale of HCC samples are also needed to retrain our CNN‐based models and validate our findings.

## CONFLICT OF INTEREST

The authors declare no conflict of interest.

## Supporting information

Supporting information

## Data Availability

Data are available upon reasonable request. The datasets used and/or analyzed during the current study are available from the corresponding author on reasonable request.
