# Supplementary material for: Deep learning‐based classification and mutation prediction from histopathological images of hepatocellular carcinoma
Source: Clin Transl Med. 2020 Jun 14;10(2):e102. doi: 10.1002/ctm2.102 (PMC7403820; doi:10.1002/ctm2.102)
Supplement: Supplementary file 1 — Supporting information [file CTM2-10-e102-s001.docx]

Supplementary Methods

**Information of somatic mutations**

The whole exome sequencing (WES) data of TCGA dataset was also downloaded from GDC data portal by searching for Data Type == "Masked Somatic Mutations". The Data Category is "Simple Nucleotide Variation". Filtering based on that, 4 files per cancer type/project will be found (one for each mutation caller), and mutation information from “mutect” were selected in our studies, in which silent mutations were filtered out. For each gene, samples with the presence of mutations (SNP/INDEL) were labelled as “mutated”. The WES data of WCH dataset were obtained from paired-end sequencing performed on an Illumina HiSeq X Ten using SureSelectXT Human All Exon V6 (target size 60 Mb, Agilent, Santa Clara, CA) according to the manufacturer’s instructions. The quality control and preprocessing of raw data (FASTQ format) was performed by using fastp(1). The reads were aligned to human hg19 genome reference using BWA (version 0.7.5a) and the BAM files were generated using SAMtools (version 1.3).

**Image preprocessing**

By using OpenSlide library(2, 3), each WSI or TMA dot stored in .svs format was extracted at magnifications of 5× and 20×, and was tiled into non-overlapping 256×256 pixel windows in .jpeg format (Fig 1C). Tiles with over 12.5% of the surface covered by background (defined as pixels with mean value > 220 in the RGB color space) were excluded at this stage. After image tiling, we normalized the R, G and B values of each tile in the respective channels by using the equation:

$$z_{i}=\frac{x_{i}-\mu}{\sigma}$$

where $x_{i}$ is the value of pixel $i$ in this channel, $\mu$ is the mean of all the pixel values in this channel, $\sigma$ is the standard error of $\mu$, and $z_{i}$ is the normalized value of pixel $i$.

**Architecture of the convolutional neural network**

Basically, the goal of our study consists of two parts (Fig 1): one is to automatically distinguish HCC from adjacent normal tissues (task 1), and the other is to predict the somatic mutations of HCC (task 2). For each task, we trained a CNN with introducing the structure of deep residual learning (4) to overcome the degradation problem. The architecture of our CNN models can be found in Fig S1. Briefly, the initial three blocks consist of two 3×3 convolutional kernels, each of which is followed by a BatchNormalization layer(5) and then a ReLU layer(6). The output of each block consists of residual from the previous block and output of the last convolutional layer in this block, which is followed by filters concentrating and ends with 1 average pooling operation. The output of the last block is subsequently inputted to a convolutional layer, a global average pooling layer, a dense layer and then a softmax output layer.

**Training and testing the convolutional neural networks**

For task 1, we used the pathological report obtained from TCGA dataset as the gold standard, and the training processes were run for 15,000 iterations (Fig 1d, left panel). The cross-entropy loss function was computed on the training set, with learning rate starting at 2e-4. During the training processes, each tile was rotated by 0, 90, 180 and 270 degree for data augmentation, in which eight 248×248 tiles from each 256×256 tile were randomly selected as the inputs. The Ranger optimization algorithm (7, 8) was used for adaptive learning rate optimization. The number of samples propagated in each batch is 64. For task 2, we trained another CNN model for each gene alteration, in which the final softmax layer of the network was replaced with a sigmoid layer to allow each single tile to be assigned with several binary labels (mutated or not), thus constructing a classifier for the mutation of each gene (Fig 1d, left panel). To ensure that there were enough WSIs for mutation prediction, we only selected the mutations of 10 most commonly mutated genes among the available tumors at this stage (Table S3). Finally, the established models (task 1 & 2) were tested on TCGA test set (Fig 1d, right panel). To challenge the trained models for task 1 & 2 and identify their limitations, TMAs from WCH dataset, in which somatic mutation (SNP/INDEL) data were available in 78 HCC samples, was used as an external validation set (Fig 1D, right panel). To evaluate the robustness of our models, a 5-fold cross-validation was performed using the WSIs from TCGA dataset.

**Statistical analysis**

The two-tailed Mann-Whitney U-test was used for numerical variables, and Fisher’s exact test was used for categorical variables. We assessed the association between the predicted probabilities (task 1) at 5× and 20× magnifications with Spearman’s correlation coefficient. The ‘plotROC’ and ‘ggplot2’ packages were used to plot the receiver operating characteristic (ROC) curves for diagnostic classification and mutation prediction. We used R version 3.6.2 to perform all the statistical tests in this study, and a two-sided *P* < 0.05 was considered statistically significant.

Supplementary Figure Legends

**Figure S1. Architecture of the CNN model.**

**Figure S2. Distribution of the number of tiles per slide (or dot). a** Distribution of the number of tiles per slide at 5× (left panel) and 20× (right panel) magnification in the test set. **b** Distribution of the number of tiles per slide at 5× (left panel) and 20× (right panel) magnifications in the external validation set.

**Figure S3. Per-tile classification probability distinguished HCC from adjacent normal samples. a** Distribution of the classification probability of HCC diagnosing per tile at 5× (left panel) and 20× (right panel) magnifications in the test set. **b** The ROC curve of per-tile classification model (AUC with 95% CI) for classifying HCC and adjacent normal tissues in the test set. **c** Distribution of the classification probability of HCC diagnosing per tile at 5× (left panel) and 20× (right panel) magnifications in the external validation set. **d** The ROC curve of per-tile classification model (AUC with 95% CI) for classifying HCC and adjacent normal tissues in the external validation set.

**Figure S4. Classifications of presence of HCC in both test set and external validation set. a, b** the ROC curves of our classification model (AUC with 95% CI) for classifying HCC and adjacent normal tissues in the test set (**a**, left panel) and external validation set (**b**, left panel), along with box plots (Mann-Whitney U-test) demonstrating the probability of HCC diagnosis predicted (right panel). The probability of each slide (or dot) was generated using Method 2. **c, d** Scatter plots showing the correlation between the per-slide classification results obtained at different resolutions (5× vs. 20×) in the test set (**c**) and external validation set (**d**). The probability of each slide (or dot) was generated using Method 2. **e, f** Bar plots demonstrating the consistency in the classification results between two magnifications (5× vs. 20×) when using binary classifiers in the test set (**e**) and external validation set (**f**). The probability of each slide (or dot) was generated using Method 2.

**Figure S5. Relationship between the number of tiles and the accuracy of the classification model** **in the test set (a-b) and external validation set (c-d)**. Rho with *P* value is shown for the linear fit obtained by Spearman’s correlation (blue line). For each figure, the upper panel represents the results aggregated by Method 1, while the lower panel represents the results aggregated by Method 2.

**Figure S6. Per-tile results of mutation prediction. a** Distribution of probability of mutation in genes from HCC tiles of WSIs in the test set, where each mutation is present or absent. ***, *P* < 0.001. **b** ROC curves associated with **a. c** Distribution of probability of mutation in genes from HCC tiles of TMA dots in the external validation set, where each mutation is present or absent. ***, *P* < 0.001. **d** ROC curves associated with **c.**

**Figure S7. Gene mutation prediction results by by counting the percentage of tiles properly classified (Method 2). a** Distribution of probability of mutation in genes from HCC tiles of WSIs in the test set, where each mutation is present or absent. ns, not significant; *, *P* < 0.05; **, *P* < 0.01; ***, *P* < 0.001. **b** ROC curves associated with **a. c** Distribution of probability of mutation in genes from HCC tiles of TMA dots in the external validation set, where each mutation is present or absent. ns, not significant; *, *P* < 0.05; **, *P* < 0.01; ***, *P* < 0.001. **d** ROC curves associated with **c.**

**Figure S8. Probability distribution on wild-type (left panel) and mutated (right panel) tiles for the predictable mutations by Method 2 in the test (a) and external validation (b) set, with average values in dotted lines.**

**Figure S9. Evolution of train loss and validation accuracy during the training**

**of the inception v3 and our model for task 1.**

**Figure S10. Evolution of train loss and validation accuracy during the training**

**of the inception v3 and our model for task 2.**

**Figure S1**


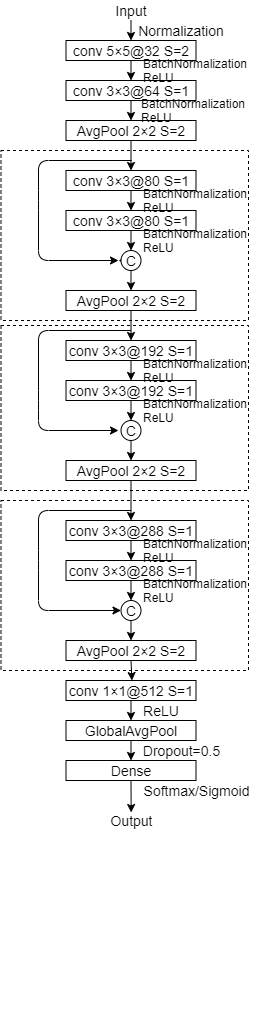


**Figure S2**


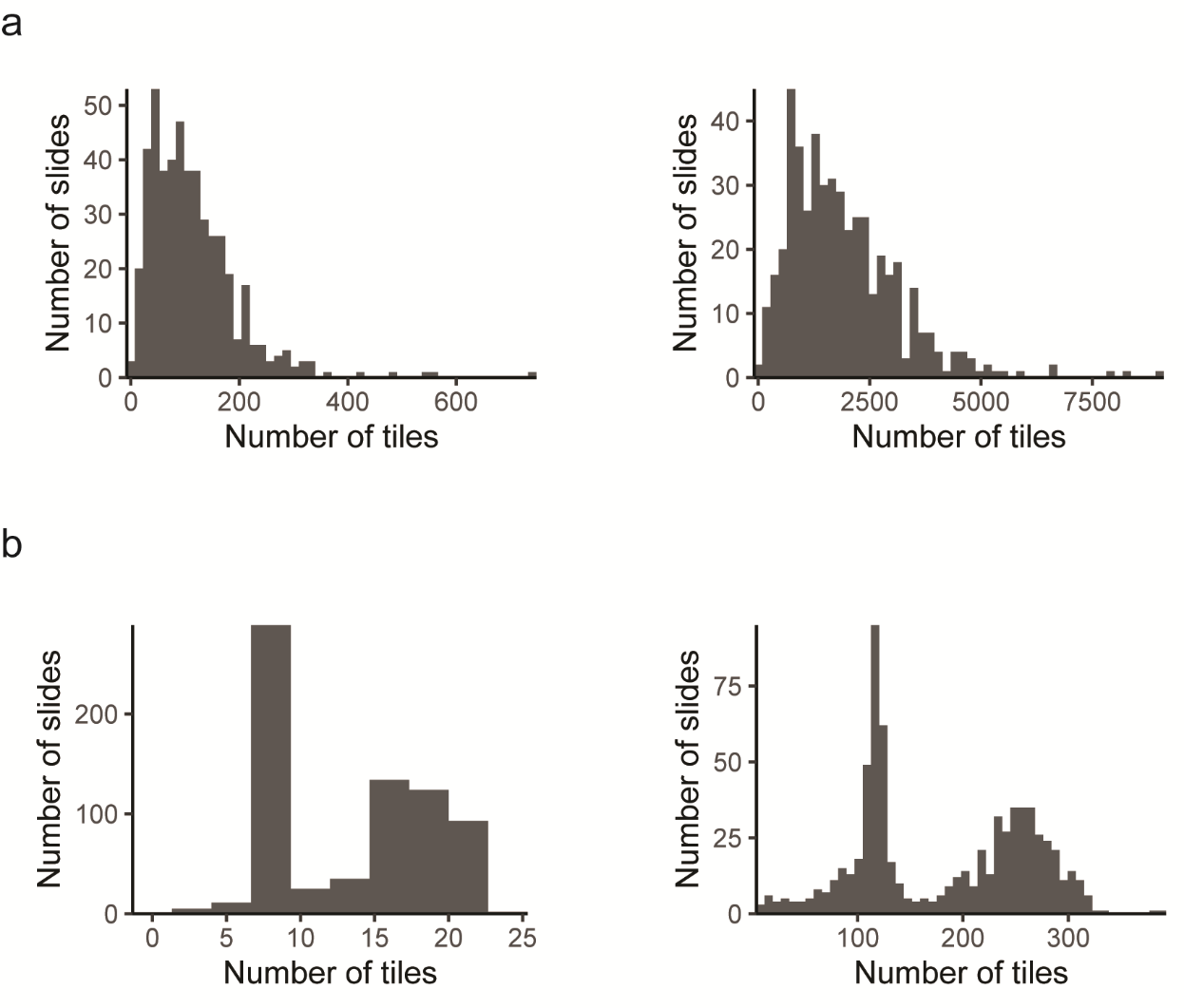


**Figure S3.**

**
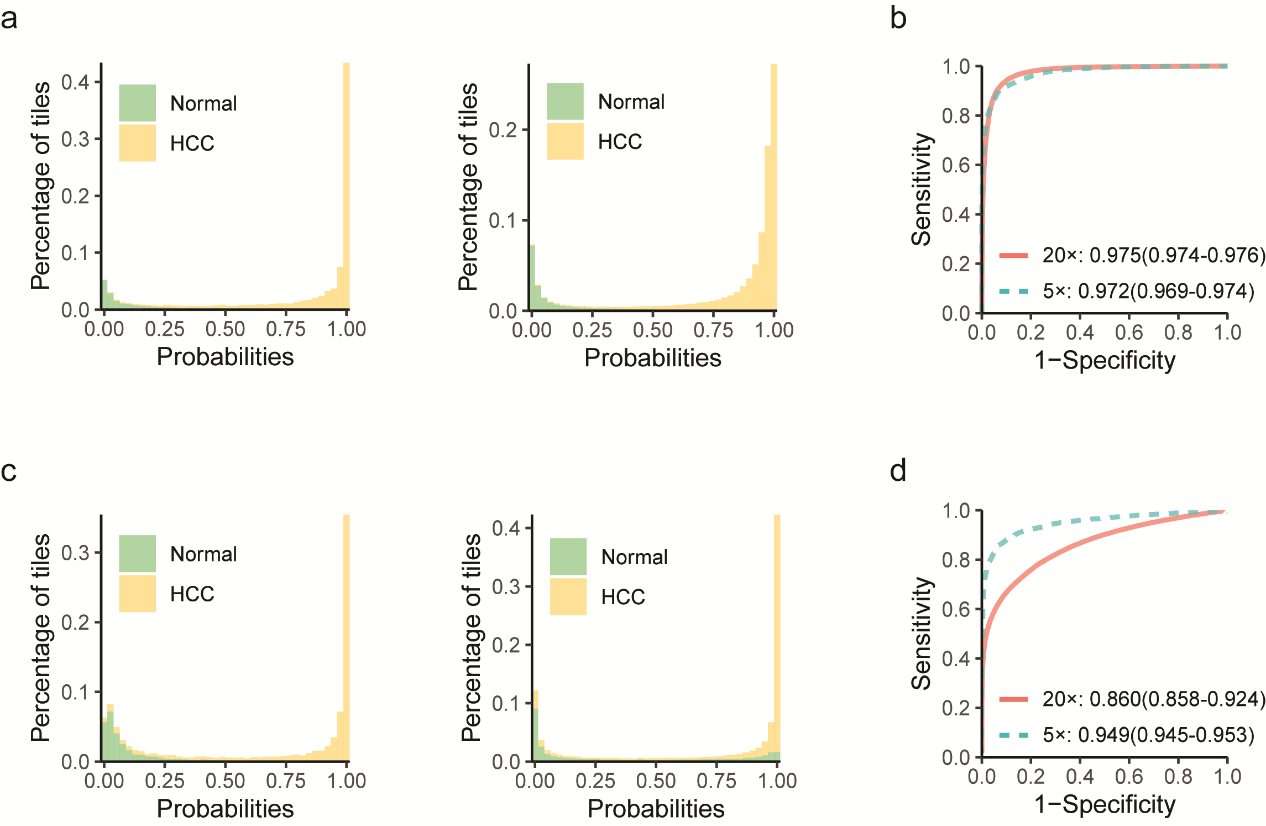
**

**Figure S4.**

**
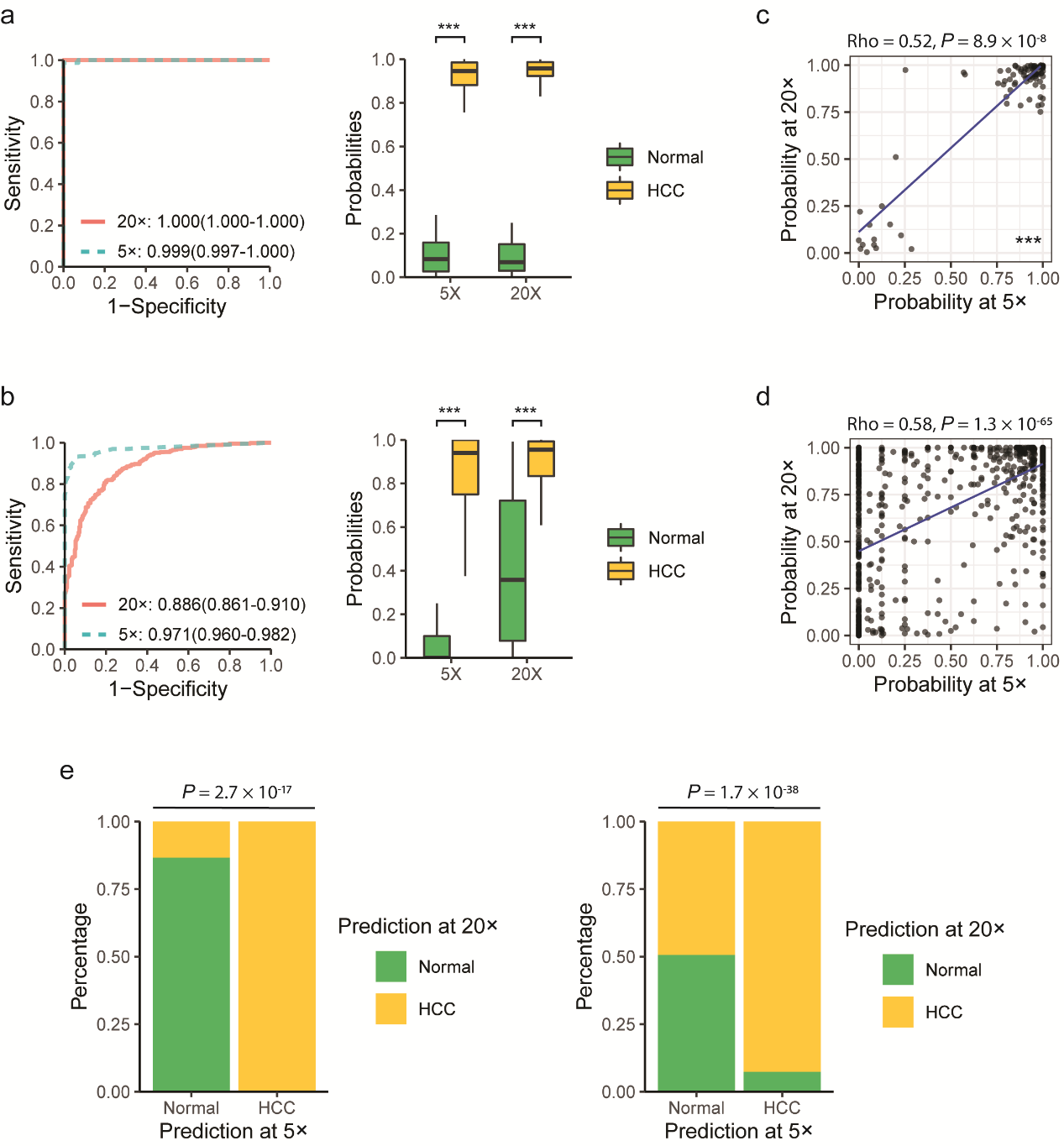
**

**Figure S5.**

**
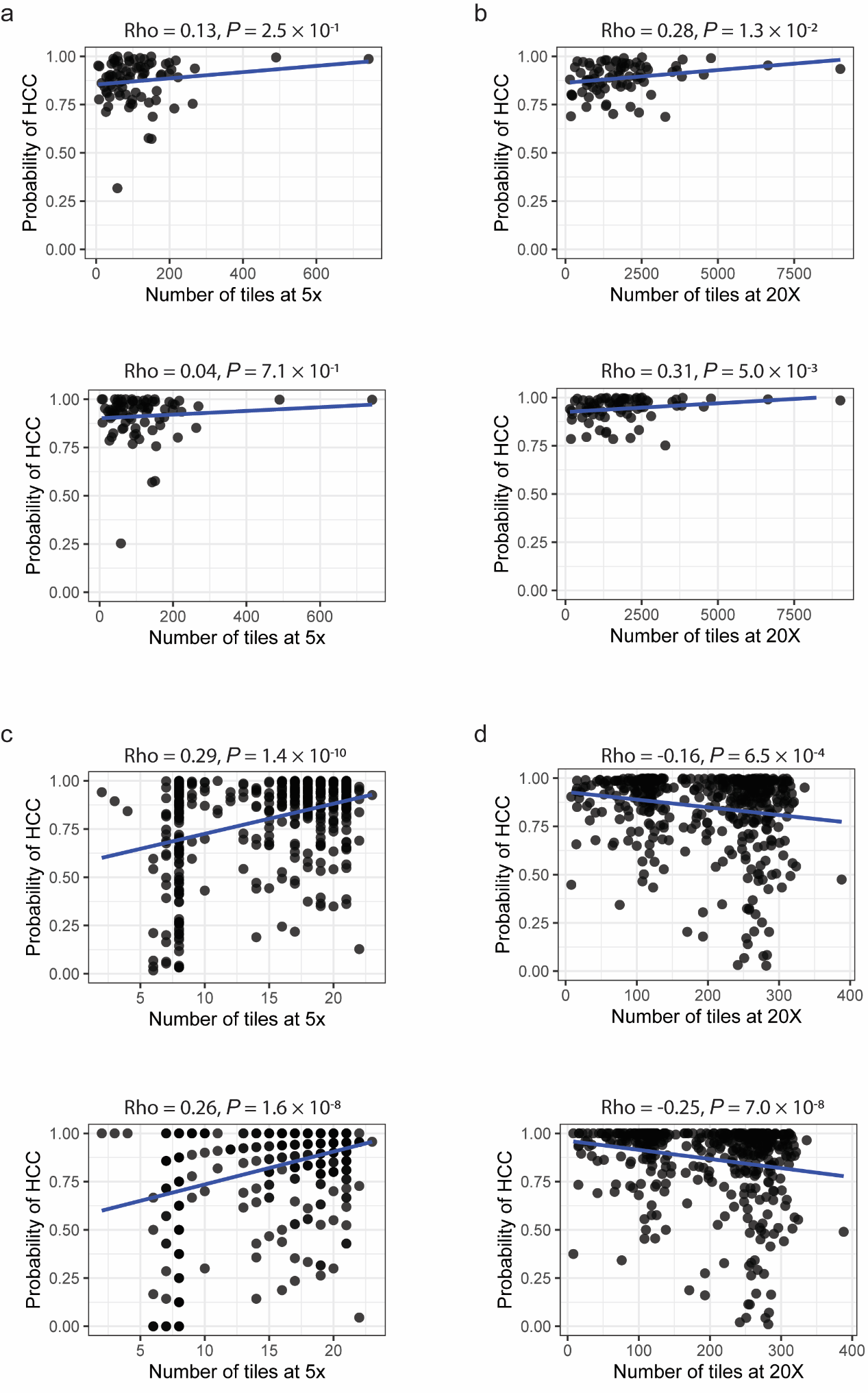
**

**Figure S6.**


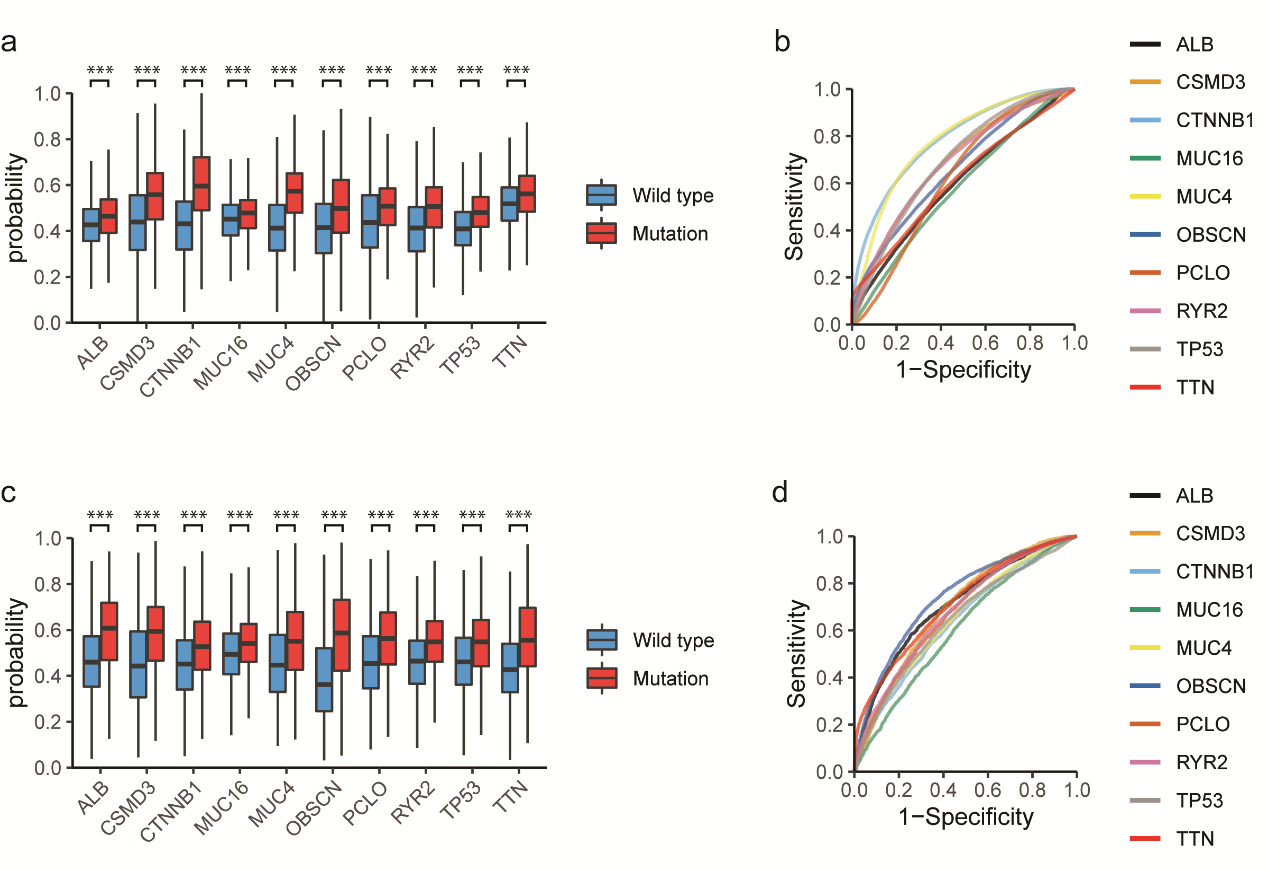


**Figure S7.**


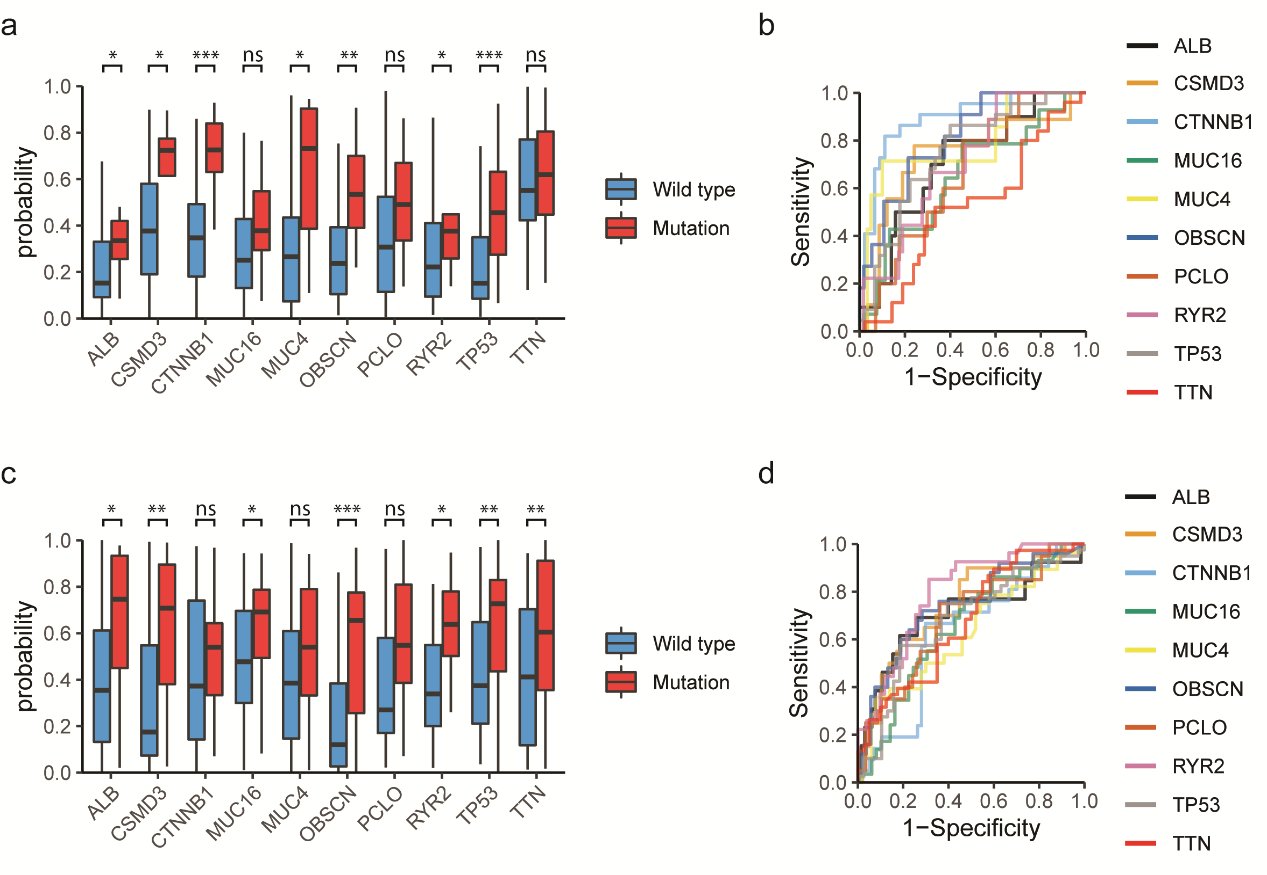


**Figure S8.**

**
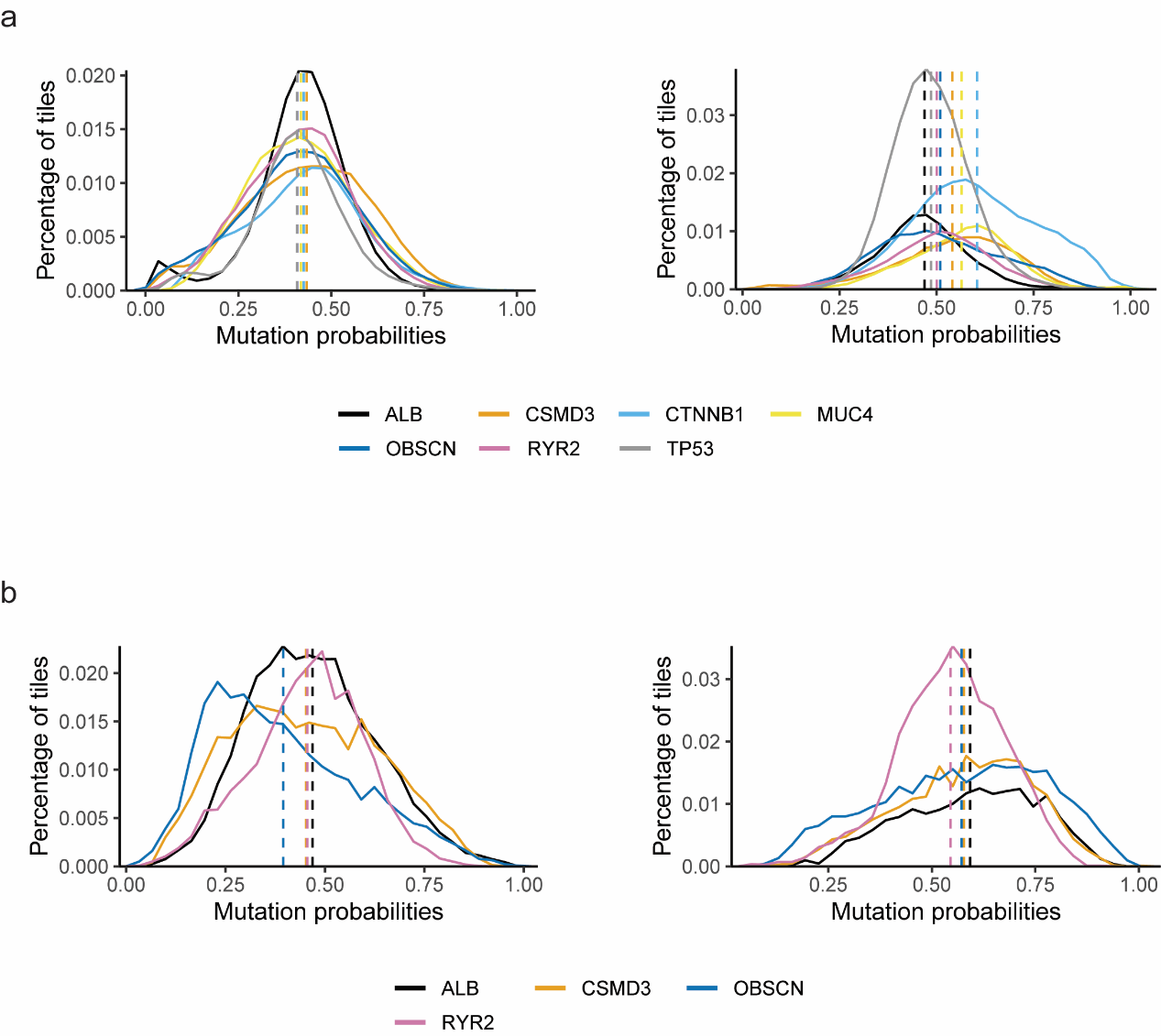
**

**Figure S9.**


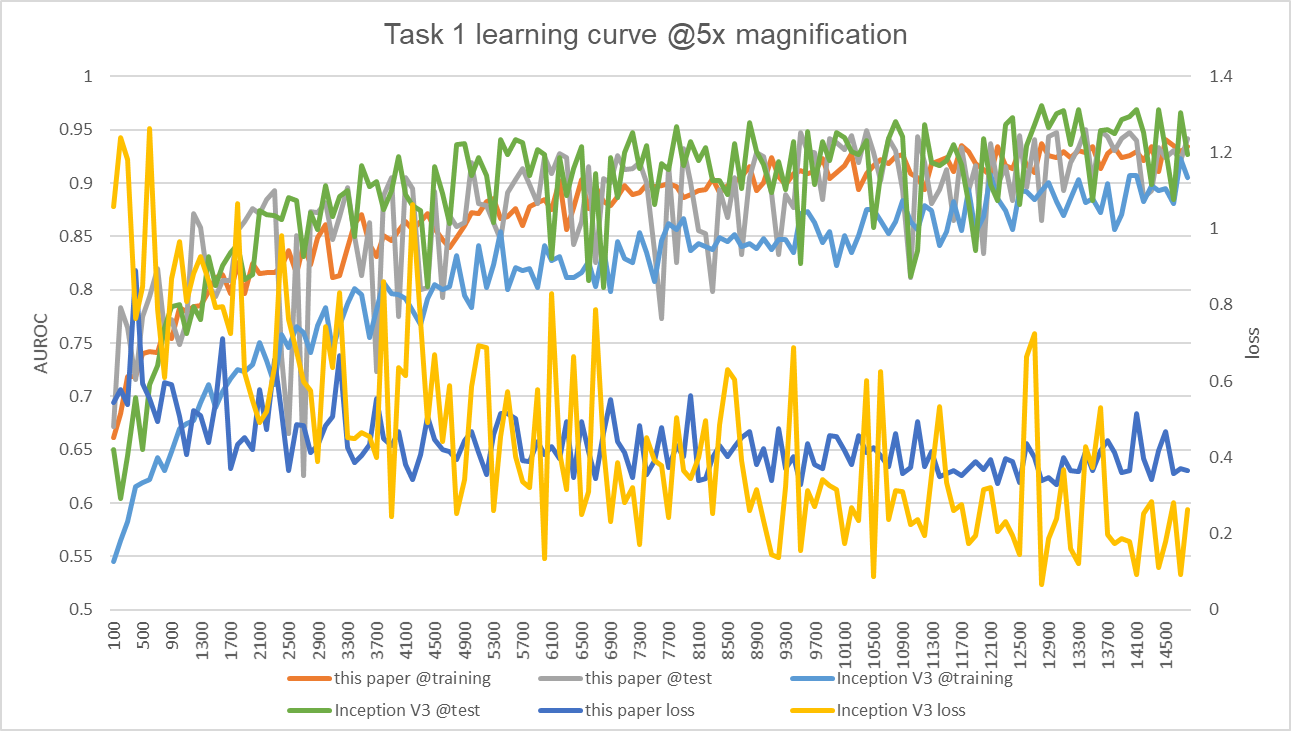


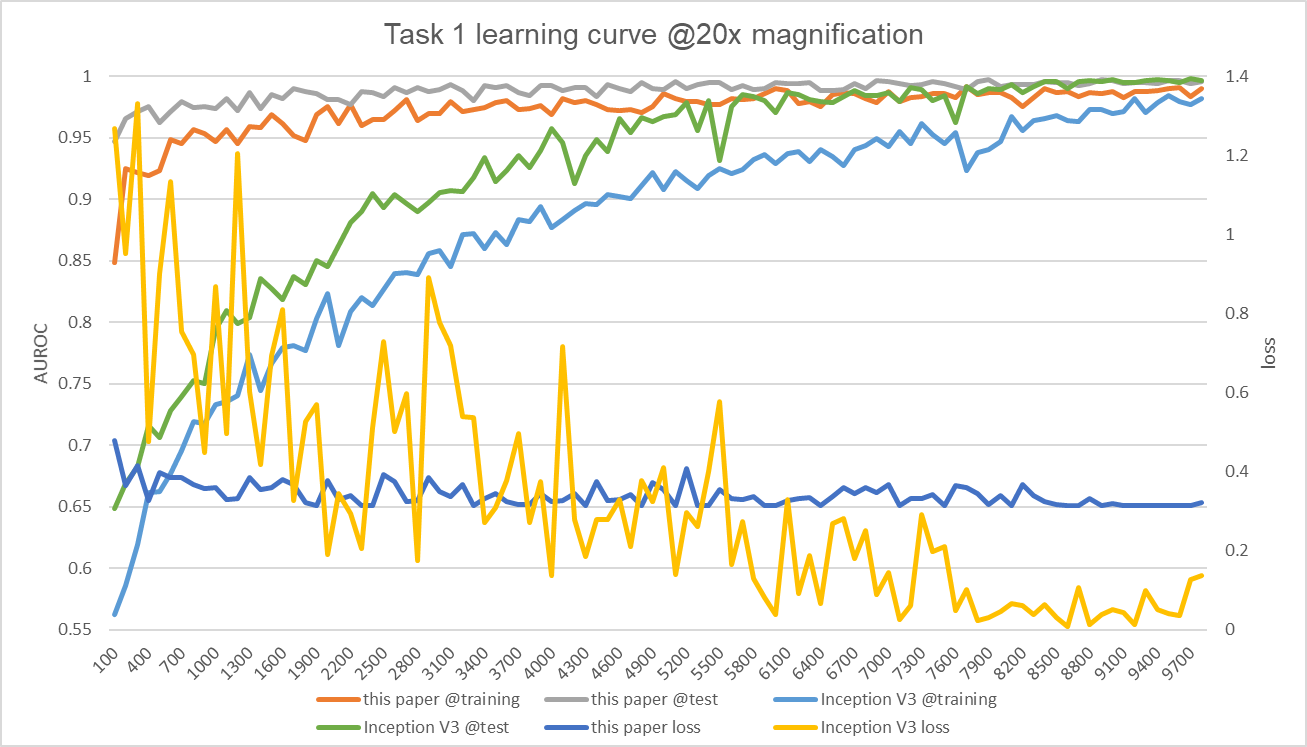


**Figure S10.**


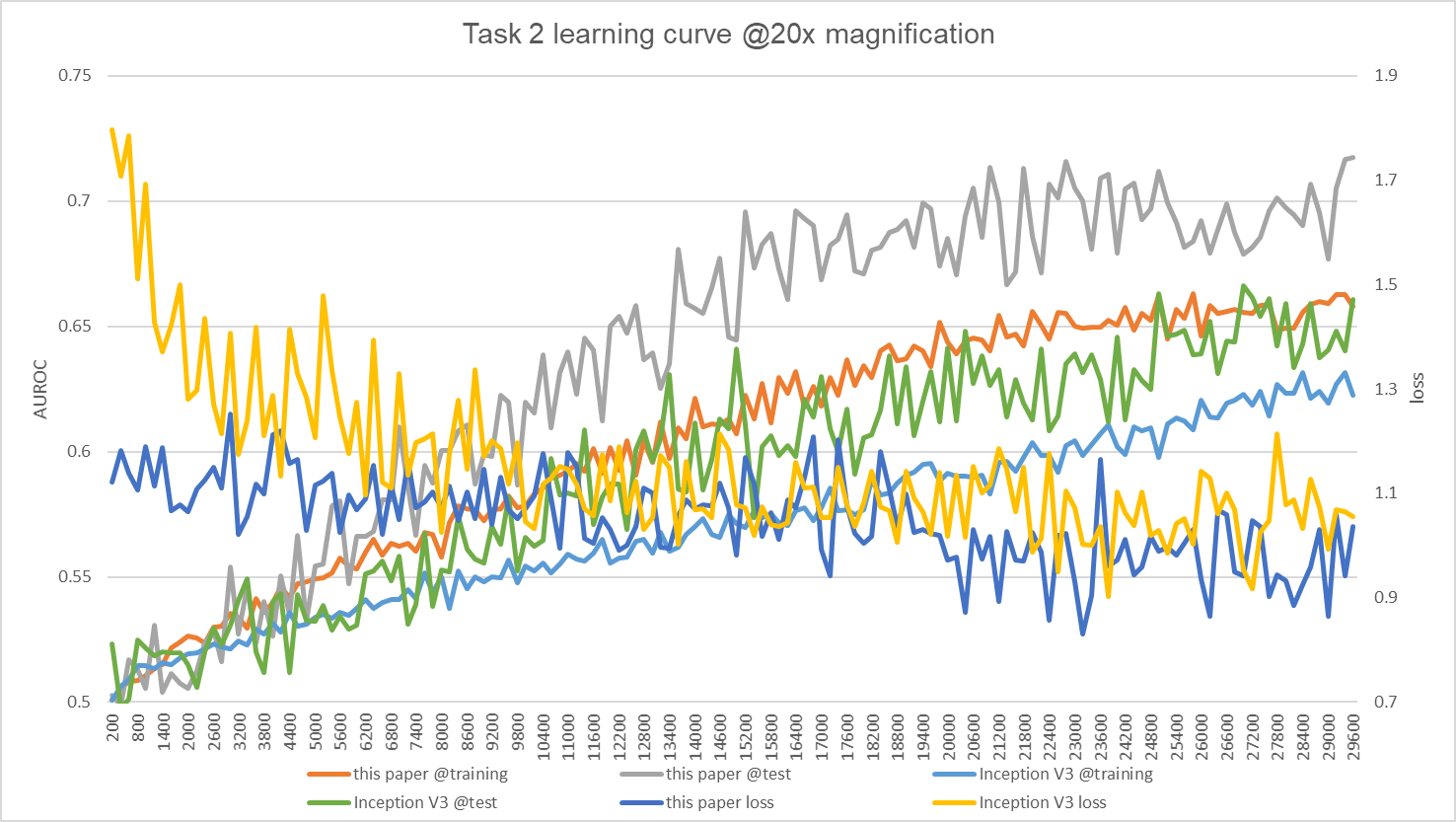


Supplementary Tables

**Table S1. Dataset information for normal vs tumor classification (number of tiles / WSIs or TMA dots in each set)**

|  | **Training** | **Test** | **External validation** |
| --- | --- | --- | --- |
| **Normal** | 179692/74 | 36885/14 | 37516/264 |
| **HCC** | 598567/314 | 155106/79 | 82979/455 |

| **Table S2. AUCs for task 1 (with 95% CIs)** | | | | |
| --- | --- | --- | --- | --- |
| **Dataset** | **Magnification** | **Tile-level** | **Per-slide AUC after aggregation** | |
|  |  |  | **Method 1** | **Method 2** |
| **TCGA** | **5x** | 0.972(0.969-0.974) | 0.998(0.994-1.000) | 0.999(0.997-1.000) |
|  | **20x** | 0.975(0.974-0.976) | 1.000 | 1.000 |
| **WCH** | **5x** | 0.949(0.945-0.953) | 0.972(0.961-0.983) | 0.971(0.960-0.982) |
|  | **20x** | 0.860(0.858-0.861) | 0.902(0.880-0.924) | 0.886(0.861-0.910) |

**Table S3. Gene included in the multi-output classification and the percentage of patients in the each database where the genes are mutated.**

| **Gene mutated** | **ALB** | **CSMD3** | **CTNNB1** | **MUC16** | **MUC4** | **OBSCN** | **PCLO** | **RYR2** | **TP53** | **TTN** |
| --- | --- | --- | --- | --- | --- | --- | --- | --- | --- | --- |
| Test set (%) | 11.3 | 10.2 | 24.2 | 16.8 | 10.2 | 10.2 | 11.5 | 10.2 | 29.1 | 28.8 |
| External validation set (%) | 16.7 | 25.6 | 26.9 | 37.2 | 35.9 | 32.1 | 25.6 | 34.6 | 51.3 | 48.7 |

**Table S4. Dataset information for mutation prediction (number of tiles / WSIs or TMA dots in each set)**

| **Training** | **Test** | **External validation** |
| --- | --- | --- |
| 569236/309 | 105310/65 | 9853/78 |

| **Table S5. AUCs for task 2 trained on mutations in the test set (with 95% CIs)** | | | |
| --- | --- | --- | --- |
|  | **Per-tile ROC** | **Per-slide AUC after aggregation** | |
|  |  | **Method 1** | **Method 2** |
| **ALB** | 0.600(0.596-0.605) | 0.686(0.473-0.899) | 0.709(0.538-0.880) |
| **TTN** | 0.613(0.610-0.616) | 0.519(0.371-0.668) | 0.521(0.375-0.667) |
| **CSMD3** | 0.687(0.682-0.691) | 0.743(0.529-0.957) | 0.739(0.535-0.944) |
| **CTNNB1** | 0.785(0.782-0.788) | 0.903(0.823-0.983) | 0.892(0.808-0.976) |
| **OBSCN** | 0.662(0.658-0.666) | 0.823(0.695-0.951) | 0.812(0.684-0.939) |
| **PCLO** | 0.624(0.621-0.628) | 0.649(0.486-0.812) | 0.658(0.495-0.821) |
| **TP53** | 0.692(0.689-0.695) | 0.773(0.657-0.889) | 0.749(0.628-0.871) |
| **MUC16** | 0.580(0.576-0.583) | 0.640(0.471-0.809) | 0.650(0.481-0.819) |
| **MUC4** | 0.777(0.773-0.781) | 0.810(0.604-1.000) | 0.786(0.570-1.000) |
| **RYR2** | 0.686(0.681-0.690) | 0.678(0.493-0.864) | 0.709(0.546-0.871) |

| **Table S6. AUCs for task 2 trained on mutations in the external validation set**  **(with 95% CIs)** | | | |
| --- | --- | --- | --- |
|  | **Per-tile ROC** | **Per-slide AUC after aggregation** | |
|  |  | **Method 1** | **Method 2** |
| **ALB** | 0.709(0.696-0.722) | 0.731(0.562-0.900) | 0.708(0.521-0.894) |
| **TTN** | 0.714(0.704-0.724) | 0.688(0.570-0.805) | 0.674(0.554-0.793) |
| **CSMD3** | 0.694(0.683-0.706) | 0.753(0.633-0.874) | 0.737(0.608-0.867) |
| **CTNNB1** | 0.642(0.630-0.654) | 0.627(0.491-0.764) | 0.622(0.484-0.761) |
| **OBSCN** | 0.741(0.731-0.752) | 0.740(0.613-0.866) | 0.740(0.617-0.863) |
| **PCLO** | 0.677(0.665-0.688) | 0.718(0.595-0.842) | 0.688(0.550-0.825) |
| **TP53** | 0.646(0.635-0.657) | 0.688(0.567-0.808) | 0.678(0.556-0.800) |
| **MUC16** | 0.605(0.594-0.617) | 0.652(0.526-0.779) | 0.653(0.529-0.778) |
| **MUC4** | 0.653(0.642-0.664) | 0.627(0.492-0.763) | 0.626(0.491-0.760) |
| **RYR2** | 0.680(0.670-0.691) | 0.797(0.700-0.895) | 0.796(0.697-0.896) |

| **Table S7. Model comparison** | | | | | | |
| --- | --- | --- | --- | --- | --- | --- |
|  | | | Our model | | Inception V3 | |
| conv/dense layers *a | | | 10 | | 33 | |
| parameters | | | 2.65M | | 21.79M | |
| FLOPs | | | **2.28G** | | 3.51G | |
| memory cost *b | | | **3.24GB** | | 9.11GB | |
| time cost *c | | | **58.12ms** | | 440.72ms | |
| metrics | | | tile-level | slide-level *d | tile-level | slide-level *d |
| Task1 AUC | 5.0 | Test set^#^ | **0.9419** | **0.9937** | 0.9268 | 0.9801 |
|  |  | External validation set^#^ | **0.9251** | **0.9856** | 0.8701 | 0.9126 |
|  | 20.0 | Test set^#^ | 0.9961 | **1** | **0.9963** | **1** |
|  |  | External validation set^#^ | **0.981** | **0.9974** | 0.9762 | 0.9973 |
| Task2 AUC *e | 20.0 | Test set^#^ | **0.7172** | **0.8122** | 0.6608 | 0.7111 |
|  |  | External validation set^#^ | **0.6786** | **0.7448** | 0.6024 | 0.6423 |

| *a | multi-size filters in one block are considered as one layer |
| --- | --- |
| *b | per batch |
| *c | per batch, during training (forwards + backwards) |
| *d | slide predicted by average of tile-level prediction |
| *e | average of 10 mutations |
| # | random results from 5-fold cross-validation |

**Reference**

1. Chen S, Zhou Y, Chen Y, Gu J. fastp: an ultra-fast all-in-one FASTQ preprocessor. Bioinformatics. 2018;34(17):i884-i90.

2. Goode A, Gilbert B, Harkes J, Jukic D, Satyanarayanan M. OpenSlide: A vendor-neutral software foundation for digital pathology. Journal of pathology informatics. 2013;4:27.

3. Goode A, Satyanarayanan M. A Vendor-Neutral Library and Viewer for Whole-Slide Images. Computer Science Department, Carnegie Mellon University, Technical Report CMU-CS-08-136. 2007.

4. He K, Zhang X, Ren S, Sun J. Deep Residual Learning for Image Recognition. 2016 IEEE Conference on Computer Vision and Pattern Recognition (CVPR). 2015:770-8.

5. Ioffe S, Szegedy C. Batch Normalization: Accelerating Deep Network Training by Reducing Internal Covariate Shift. 2015.

6. Glorot X, Bordes A, Bengio Y. Deep Sparse Rectifier Neural Networks2010.

7. Zhang MR, Lucas J, Hinton G, Ba J. Lookahead Optimizer: k steps forward, 1 step back.

8. Liu L, Jiang H, He P, Chen W, Liu X, Gao J, et al. On the Variance of the Adaptive Learning Rate and Beyond. 2019;abs/1908.03265.
